# Notice to Medical Men in Illinois

**Published:** 1860-08

**Authors:** F. K. Bailey, R. G. Laughlin, H. R. Payne

**Affiliations:** Chairman, Joliet, Will Co.; Heyworth, McLean Co.; Marshall, Clark Co.


### NOTICE TO MEDICAL MEN IN ILLINOIS.

The undersigned were appointed at the last meeting of the Illinois State Medical Society, a Committee on Drugs and Medicines.

In the constitution of the Society we find the duties of the Committee to be as follows:

"The Committee on Drugs and Medicines shall prepare an annual report on all improvements and discoveries in pharmacy and materia medica, effected during the year, and on all subjects connected with the sophistication and sale of drugs and medicines."

The Committee would respectfully request the physicians of this State to report, in reference to this subject, the results of their observations, and send as soon as the first of April, 1861, to

F. K. BAILEY, Chairman,
Joliet, Will Co.
R. G. LAUGHLIN,
Heyworth, McLean Co., or
H. R. PAYNE,
Marshall, Clark Co.

